# Evaluation of endoscopic vein extraction on structural and functional viability of saphenous vein endothelium

**DOI:** 10.1186/1749-8090-6-82

**Published:** 2011-06-10

**Authors:** Bader E Hussaini, Xiu-Gui Lu, J Alan Wolfe, Hemant S Thatte

**Affiliations:** 1 Cardiothoracic Surgery Division, Veterans Affairs Boston Healthcare System, Boston, MA, USA; 2 Brigham and Women's Hospital, Boston, MA, USA; 3 Harvard Medical School, Boston, MA, USA; 4 Saint Joseph's Hospital of Atlanta, Atlanta, GA, USA

**Keywords:** CABG venous grafts, Endoscopic procedures, eNOS, Nitric Oxide, Vascular reactivity

## Abstract

**Objectives:**

Endothelial injury during harvest influences graft patency post CABG. We have previously shown that endoscopic harvest causes structural and functional damage to the saphenous vein (SV) endothelium. However, causes of such injury may depend on the extraction technique. In order to assess this supposition, we evaluated the effect of VirtuoSaph endoscopic SV harvesting technique (VsEVH) on structural and functional viability of SV endothelium using multiphoton imaging, biochemical and immunofluorescence assays.

**Methods:**

Nineteen patients scheduled for CABG were prospectively identified. Each underwent VsEVH for one portion and "No-touch" open SV harvesting (OSVH) for another portion of the SV. A two cm segment from each portion was immersed in GALA conduit preservation solution and transported overnight to our lab for processing. The segments were labeled with fluorescent markers to quantify cell viability, calcium mobilization and generation of nitric oxide. Morphology, expression, localization and stability of endothelial caveolin, eNOS, von Willebrand factor and cadherin were evaluated using immunofluorescence, Western blot and multiphoton microscopy (MPM).

**Results:**

Morphological, biochemical and immunofluorescence parameters of viability, structure and function were well preserved in VsEVH group as in OSVH group. However, tonic eNOS activity, agonist-dependent calcium mobilization and nitric oxide production were partially attenuated in the VsEVH group.

**Conclusions:**

This study indicates that VirtuoSaph endoscopic SV harvesting technique preserves the structural and functional viability of SV endothelium, but may differentially attenuate the vasomotor function of the saphenous vein graft.

**Ultramini-Abstract:**

Endoscopic extraction preserved the structure and function, but attenuated the calcium mobilization and nitric oxide generation in human SV endothelium.

## Introduction

The Saphenous Vein (SV) is the most commonly used autologous homograft in patients undergoing coronary artery bypass grafting (CABG) surgery. Preserving viable endothelium of SV grafts harvested during coronary revascularization impacts the grafts patency rate [1-11]. The integrity of the endothelial lining is affected by many factors during harvest. Using epifluorescence multiphoton imaging techniques, we have previously shown that the pH, temperature, SV distention, and composition of storage solution can affect the endothelial viability and function [4,12-15]. Similarly, surgical manipulation can also damage the endothelium of SV grafts, increasing the risk of vasospasm, thrombogenesis, occlusive intimal hyperplasia, and stenosis [4,5,10,11].

Saphenous vein has traditionally been harvested using an open surgical technique with minimal manipulation of the vein [16]. However, many centers have adapted to the minimally invasive surgical technique of endoscopic saphenous vein harvest (EVH) because of patient preference and decreased incidence of, lower extremity morbidity, related to cellulitis and wound infection, hematoma, seroma, edema, and saphenous neuropathy and neuralgia compared to the open technique [17-25]. The recently published secondary analysis of PREVENT-4 study compared outcomes after on-pump vs off-pump CABGs and reported SV graft occlusion rates of 46.7% at 12-18 months, far in excess of historical assumptions by cardiovascular surgeons. More concerning, they found EVH to be an independent predictor of decreased SV graft patency at one year, the cause of which was not defined, warranting further investigations [26]. Likewise, a recently completed randomized on/off bypass (ROOBY) trial has concluded that EVH was associated with lower SV graft patency at 1-year and higher rate of perioperative myocardial infarction and need for revascularization within 1-year compared to OSVH [27]. Although both the PREVENT IV and ROOBY studies were not randomized with regard to the SV harvest technique, their findings are of great concern due to the large number of cases that were evaluated.

Although the EVH procedures utilizing the currently available technology follow a similar minimally invasive technique, differences still exist due to the unique properties of different devices as well as the experience of surgical operator. This study design evaluates viability of veins harvested by the VirtuoSaph endoscopic vein harvesting technique and OSVH methodology. The OSVH consisted of removing the saphenous vein with perivascular tissue in the traditional open fashion with "No-touch" technique. We compared the viability and functionality of SV endothelium between VsEVH, and OSVH as an internal control, using three independent techniques: 1) epifluorescence multiphoton microscopy (MPM); 2) immunofluorescence and; 3) biochemical assays.

## Materials and methods

### Study Design and Experimental Protocol

This study was designed to be consistent with our earlier study [28]. Patients, ages 56 - 82, (average 69.3 years), scheduled for elective coronary artery bypass surgery at the Saint Joseph's Hospital of Atlanta, were prospectively identified for the evaluation of VirtuoSaph endoscopic harvest instrumentation and technique. The vein samples were collected according to the protocol approved by the Human Studies Subcommittee, and after obtaining informed consent from the patients. Each patient underwent VsEVH for the proximal portion of the vein and OSVH for the distal portion of the vein. Endoscopic incision was located just below the knee, with "No touch" incision in the upper calf; samples were taken to include side branches in every instance. At no time was the vein insufflated with any solution, and the "experimental" endoscopic sample was obtained from the midportion of the thigh segment after exteriorization to accommodate prioritized patient requirements for a suitable bypass conduit. For the VsEVH portion, minimal CO_2 _insufflation using an open system was used for visualization and dissection of the tissues around the vein. Once the vein was mobilized, the side branches were simultaneously cut and cauterized with the bipolar V-cutter/cautery. The distance between the two sampling areas was (26.58 ± 3.84 cm).

Harvesting techniques were performed by two experienced physician's assistants with over 7 years EVH experience, and greater than 2000 cases performed, respectively. VsEVH was conducted according to Terumo guidelines and training, utilizing the VirtuoSaph Endoscopic Vein Harvesting System (MCVS550, Terumo Cardiovascular Systems Corp., Ann Arbor, Michigan). A two centimeter portions of the VsEVH and OSVH vein were immersed in ice cold GALA, and transported overnight in a CoolPack by FedEx, from Atlanta to our laboratory in VABHS, Boston, MA. Transit time and temperature (20 ± 2.2 hours; 7 ± 2.3°C; respectively) were recorded in the laboratory.

### Structural and Functional Assays

#### Cell Viability Assay

Structural and functional viability of saphenous vein endothelial and smooth muscle cells were assessed with a fluorescence-based Live-Dead (calcein AM/ethidium homodimer) assay and MPM as described [4,12-14,28,29]. The SV segments were incubated with calcein AM and ethidium homodimer dyes (10 μM, final concentration) in 1.5 ml of Hanks balanced salt solution (HBSS), pH 7.4, for 30 minutes at 21°C. After incubation, segments were washed three times with HBSS, mounted on the multiphoton microscope stage in an imaging chamber, and viable cells (green fluorescence) and damaged cells (red fluorescence) were imaged as described below.

### Measurement of Esterase Activity

The conversion of calcein AM ester (nonfluorescent) to green fluorescent calcein by the esterases in living cells was used as a marker of esterase activity in the endothelial cells of the vein segments. Saphenous vein lumen and endothelial cell layer were identified by XYZ scanning using MPM. Specifically, five different regions of uniform size were marked on the endothelial cells in the lumen of each segment using image processing software (MetaMorph Imaging Series; Universal Imaging, Corp., West Chester, PA). Total integrated fluorescence intensity (photon counting) in the marked regions of the endothelium was measured as a function of esterase activity in the SV segments using MetaMorph [4,12-14,28,29].

### Intracellular Calcium Mobilization and Nitric Oxide Generation

Calcium mobilization and nitric oxide generation in the endothelial cells of the SV was measured using calcium sensitive calcium orange dye and nitric oxide specific diaminofluorescein (DAF) dye, as previously described [4,12-14,28,29]. Resting calcium levels and basal/tonic activity of eNOS were measured in the absence of bradykinin stimulation. The segments were imaged using MPM, and bradykinin stimulated calcium mobilization and nitric oxide generation were assessed in real time over the course of 10 minutes and quantified as described below.

### Quantitative Analysis of Calcium and Nitric Oxide

Calcium mobilization and nitric oxide generation were measured by recording changes in calcium orange and DAF fluorescence using MPM imaging, before and after bradykinin treatment as described [4,12-14,28,29]. Typically, five specific regions were drawn along the endothelial region of the lumen for each vein segment using MetaMorph image processing software. Fluorescence intensity was integrated over all pixels within the boundary of each individually enclosed area and the quantum yield was measured in calcium and nitric oxide fluorescence channels, respectively. The fluorescence intensity from each image was normalized by values determined from a reference image recorded before bradykinin treatment [4,12-14,28,29].

### Immunofluorescence

Vein frozen sections were labeled with primary caveolin 1, eNOS, Cadherin and von Willebrand factor (vWF) antibodies as described [28,29]. After primary antibody labeling, sections were washed 3X with PBS and labeled with either fluorescein and/or texas red conjugated secondary antibodies (1:200 dilution) in PBS for 2 hours at 21°C. Labeled samples were washed 3X with PBS, mounted and imaged using MPM.

### Multiphoton Microscopy

Imaging and fluorescence measurements in all samples were performed with a Zeiss LSM 710 Confocal-MaiTai multiphoton imaging system (Carl Zeiss Microimaging, Inc., Thornwood, NY; Spectra-Physics, Mountain View, CA) as described previously [4,12-14,28,29] in transmission and epifluorescence mode. The 512 × 512 pixel images were collected in direct detection configuration at a pixel resolution of 0.484 μm. The endothelial cell layers were identified by XYZ scanning and imaged at depths of 50 μm away from the site of excision in transverse sections of the SV segments.

### Western Blotting

Protein extraction and electrophoresis were performed as described [28,29]. All SV samples were processed at 4°C. Twenty milligrams of SV was cut into 300 small pieces and suspended in 200 ml of CelLytic MT Lysis/Extraction buffer (Sigma) containing a protease inhibitor cocktail (Sigma). The tissue was homogenized, centrifuged and the protein concentration in the supernatant was measured using the Bio-Rad protein assay. Equal amount of total proteins (50 mg) were resolved on 7.5%, 10% or 12% SDS-PAGE, and electro-blotted onto the nitrocellulose membrane (BioRad). Blots were incubated with anti- caveolin 1, eNOS, cadherin, and vWF antibodies (1:1000) and were subsequently incubated with horseradish peroxidase conjugated secondary antibodies (1:8000; DAKO). Bound antibodies were detected using ECL (Amersham Biosciences, Sweden). The blots were imaged and analyzed using MetaMorph, [28,29].

### Statistical Analyses

Different individuals in blinded fashion performed the imaging, data extraction and analysis. Data are expressed as mean ± standard deviation unless otherwise stated. The differences between the two groups (OSVH and VsEVH) were compared using Student's t-test. Statistical significance was accepted at the 95% confidence level (P < 0.05). The data was derived from n = 475 measurements for esterase activity, and from n = 95 for calcium and nitric oxide assays, respectively, for each group investigated. All analyses were performed using SAS statistical software (version 9.2, SAS Institution, Gary, NC)

The authors had full access to the data and take full responsibility for its integrity. All authors have read and agreed to the manuscript as written. The funding agencies did not play any role in influencing data collection, extraction and interpretation.

## Results

**Figure 1:**
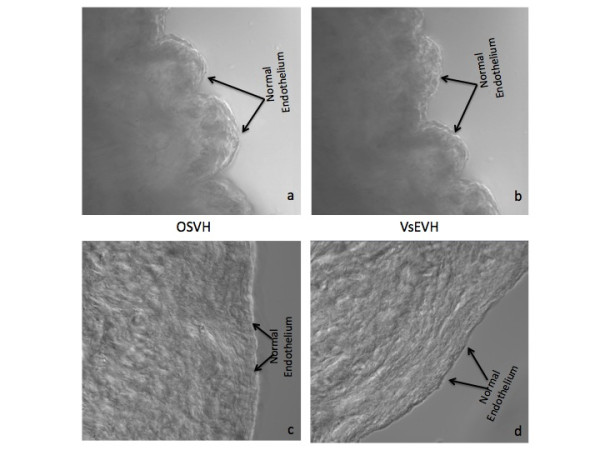
**Multiphoton images of SV in the transmission mode**. Endothelium and smooth muscle cells do not show visible damage and remain intact in vessels harvested by both techniques. OSVH: open saphenous vein harvest; VsEVH: VirtuoSaph endoscopic harvest. Magnification 400X Figure 1a. Intact saphenous vein Figure 1b. Frozen sections: 40 μ m

**Figure 2:**
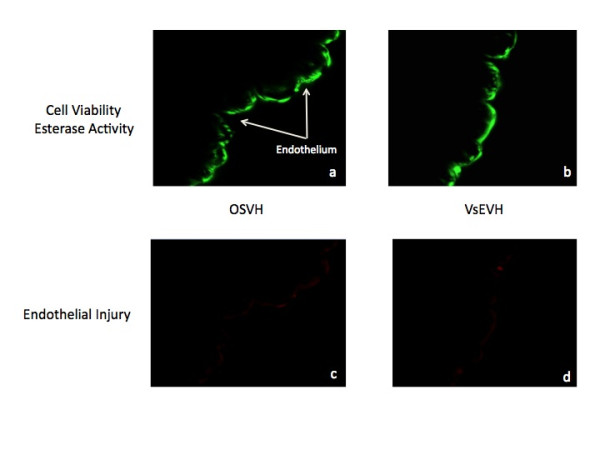
**Esterase activity in the OSVH and VsEVH samples: **Representative images showing similar esterase activity and viability (green fluorescence) in both samples (a and b). Both techniques caused minimal visible damage to the vessels as indicated by attenuated red fluorescence in endothelial and smooth vessel regions (c and d). Magnification = 400 ×.

Multiphoton imaging of SV samples in transmission mode did not reveal any stretching, detachment or gross breaks in endothelium and smooth muscle cells in both groups, Figure 1a and 1b. Similarly, morphological abnormalities were not observed in images of thin sections of SV samples in both groups, Figure 1c and 1d. The endothelium remains firmly attached to the medial region with no damage or breaks in the continuity of the structure. Endothelial cells exhibited robust green fluorescence, demonstrating structural integrity and viability in both, OSVH and VsEVH samples, Figures 2a and 2b. Additionally, vein samples in both groups did not exhibit any membrane damage, either in the endothelium or the smooth muscle cells, as indicated by minimal red fluorescence, Figures 2c and 2d. Similarly, measurement of green fluorescence quantum yield, a function of esterase activity, did not show any significant difference (p < 0.2478) between OSVH and VsEVH (186.83 +/- 49.82 vs 190.18 +/- 55.82, arbitrary units, mean +/- SD, n = 475 measurements), respectively, indicating similar endothelial cell viability.

**Figure 3:**
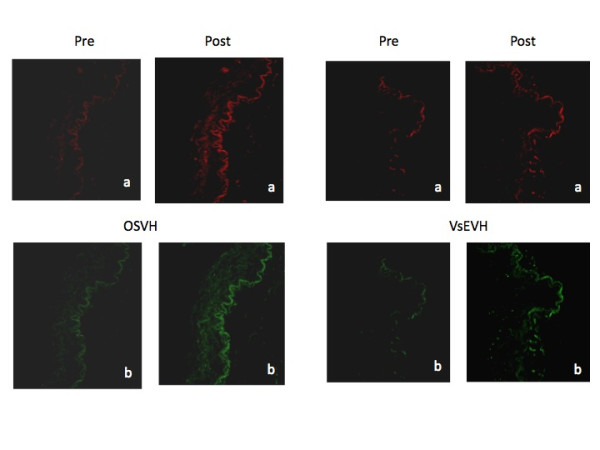
**Bradykinin mediated calcium mobilization and NO production in SV; **(A) Representative images of calcium mobilization pre and post bradykinin stimulation in the OSVH and VsEVH groups. (B) Representative images of nitric oxide production pre and post bradykinin stimulation. Magnification = 400 x.

**Figure 4:**
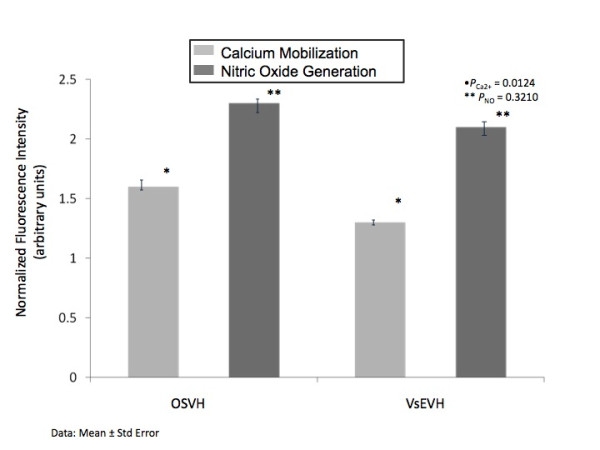
**Quantitative representation of normalized calcium mobilization and nitric oxide production in the OSVH and VsEVH groups**. Bradykinin induced calcium mobilization and nitric oxide production was greater in the OSVH group over baseline than in the VsEVH group. N = 95 measurements for each group.

Endothelial calcium and nitric oxide response in absence (tonic/basal level) and presence (stimulatory/functional response) of bradykinin are shown in Figure 3. Bradykinin stimulation resulted in increase in calcium and nitric oxide fluorescence in both groups. However, measurement of calcium mobilization and nitric oxide production (quantum yields) in OSVH and VsEVH group demonstrated a differential response. Bradykinin stimulated calcium response was significantly greater in OSVH than in VsEVH endothelium (p < 0.0124). Similarly, in response to bradykinin stimulation of eNOS, nitric oxide production in OSVH was greater than in VsEVH group, but not significantly different (p < 0.321), Figure 4.

**Figure 5:**
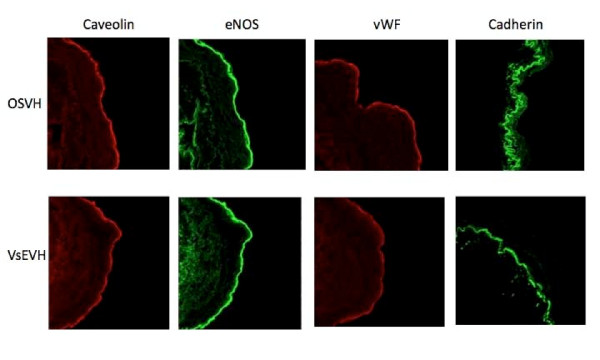
**Immunofluorescent labeling of endothelial cell markers in SV samples**. Representative fluorescence images showing normal distribution of caveolin, eNOS, von Willebrand factor and cadherin in both, OSVH and VsEVH groups. Harvesting of SV using the VirtuoSaph endoscopic technique did not damage or alter the localization of proteins in the endothelium. Magnification = 400 x.

Expression and localization of Caveolin, eNOS, vWF and cadharin, components of endothelial cells involved in cell signaling, structure and function, were similar in both, OSVH and VsEVH groups, as demonstrated by robust immunofluorescence in the endothelium of the vein samples, Figure 5. Quantitative analysis of the fluorescence did not show any significant difference between the two groups.

**Figure 6:**
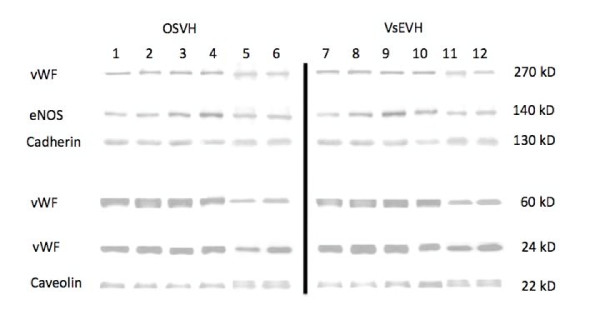
**Western blot analysis of SV harvested by OSVH and VsEVH technique demonstrates similar resolution and concentration of endothelial proteins**. Endoscopic harvesting using VirtuoSaph did not negatively affect the endothelial proteins in SV samples. Samples 1-6: OSVH Group; Corresponding samples 7-12: VsEVH Group.

Western blots of SV extracts did not demonstrate any significant difference in the resolution of proteins between OSVH and VsEVH groups, Figure 6, confirming our immunofluroescence observations, Figure 5. Even though, intra variability in protein components between patients was different, and is clearly visible on the Western blots, the inter variability between the two procedures within a patient sample was very similar, Figure 6. Therefore, averaged values of densitometer scans of proteins in OSVH samples were not different than those in the VsEVH group within the patient sample.

The measurable changes observed in structural and functional proteins between the two harvesting techniques as measured by the three different assaying techniques are summarized in Table 1.

**Table 1:** Summary of Results

Technique	OSVH compared to VsEVH
**Multiphoton Microscopy**	

Structure/morphology	**→**

Endothelial/SMC damage	**→**

Esterase Activity	**→**

Calcium Mobilization after BK stimulation	**↑**

NO production after BK stimulation	**↑**

	

**Immunofluorescence**	

Caveolin	**→**

eNOS	**→**

vWF	**→**

Cadherin	**→**

	

**Western Blot**	

Caveolin	**→**

eNOS	**→**

vWF	**→**

Cadherin	**→**

SMC = smooth muscle cells; BK = bradykinin; NO = Nitric Oxide; eNOS = Endothelial Nitric Oxide Synthase; vWF = Von Weillebrand Factor

## Discussion

Despite the widespread acceptance that arterial grafts are superior to venous conduits in coronary revascularization procedures, the desire to achieve complete revascularization and the relative ease of use of SV conduits has ensured their continued widespread usage. Most cardiovascular surgeons are well aware of the necessity to avoid over distension of the SV conduit during harvest and subsequent preparation, in preventing mechanical disruption of the endothelium. Despite our earlier communication that endovascular harvest techniques resulted in profound intimal injury relative to classic open harvest "No-touch" techniques [28], patient demand for improved cosmesis and quicker functional recovery has superseded concerns regarding endothelial integrity. Indeed, the mounting evidence that endoscopic saphenous vein harvest can reduce lower extremity morbidity, has led many surgeons to adopt this technique [20-26]. However, the endoscopic technique may result in increased mechanical traction on the vein, promotes possible thermal injury by the use of cautery to control side branches, and result in excessive exposure to an acidotic environment from high CO_2 _pressure, which may result in impaired endothelial function of the venous conduits. Therefore, development of both, adequate instrumentation and reproducible technique of endoscopic harvest to overcome these draw backs is of prime importance. We hope to continue to address these issues at the basic level, with our previous [28], current, and ongoing studies, to be able to provide informed choices for the cardiac surgeons in deciding open or endoscopic harvesting for improved conduit quality and long-term outcomes for the patients.

In this investigation, as the SVs from harvest to analysis involved substantial transit time, and cold, albeit, potentially variable temperature exposure, it was of primary concern to us that the vessels remain fully viable during this period. We have previously shown that GALA surgical conduit preservation solution, used in our hospital for CABG and peripheral vascular surgery, maintains the structural and functional viability of the blood vessel endothelium during short- and long-term (over 24 hour) storage [14]. This coupled with excellent graft patency, and long-term outcomes in over 3000 patients treated with GALA in our VA hospital convinced us that GALA would be an ideal solution for preservation and transport of the SV samples to our laboratory. That, GALA indeed preserves the morphological and physiological viability of SV conduits during transit, was reconfirmed by the fact that the vein samples remained structurally and functionally intact, with no visible morphological change, membrane damage or loss of proteins, and with robust calcium mobility, eNOS activity and NO generation, Figures 1-6. Measured values of these samples were within the range for those of freshly harvested and analyzed SVs as reported by us [4,12-14,28,29].

We have previously shown that the endothelium is substantially damaged during harvesting of SV using an endoscopic technique [28]. The parameters of endothelial structure and function were altered significantly in the endoscopic samples, including the impairment of esterase activity, calcium mobilization and nitric oxide generation. Additionally, extensive stretching and disruption of membrane proteins, demonstrating damage to the endothelium and the smooth muscle cells were also observed [28]. In contrast, in this study, harvesting of the SV using the VirtuoSaph did not reveal any structural and functional cellular damage. Morphological structure, esterase activity and endothelial viability were well maintained in the endoscopic samples (VsEVH), similar to those observed in the corresponding samples harvested by the "No touch" open technique (OSVH), Figures 1-4.

These findings were also confirmed by using two other independent assessment techniques. Immunofluorescence labeling of vessel samples demonstrated that expression, localization and distribution of caveolin, eNOS, vWF and cadharin were well preserved in VsEVH endothelium similar to those in OSVH samples, Figure 5. Equally, Western blot analysis demonstrated that the endothelial proteins were well conserved in samples from both groups, Figure 6. These results clearly demonstrate that unlike our previous observation [28], endoscopic harvesting of SV using VituoSaph does not cause structural damage to the endothelium and the smooth muscle.

Even though we did not observe any visible and measureable changes in caveolin and eNOS in the endothelium of VsEVH samples, the calcium mobilization and nitric oxide production appeared to be differentially altered. As shown in Figures 3 and 4, Bradykinin stimulated mobilization of calcium and eNOS mediated nitric oxide generation was deceased in VsEVH samples in comparison to the OSVH samples, indicating attenuation of endothelial function, confirming our earlier observation [28]. Preservation of bypass conduit eNOS activity and nitric oxide generation has important implications on immediate graft function, long-term patency and patient outcomes. Because nitric oxide induces vasodilation, inhibits platelet and neutrophil adhesion and prevents atherosclerosis, the impaired ability of the endoscopically harvested SV endothelium to produce nitric oxide, may lead to attenuated vasomotor function with significant implications on graft patency and patient outcomes. It is not clear, however, that the impairment we observed in the endothelial response is permanent or of clinical significance. It is possible that this defect may reverse with time, especially as VsEVH samples do not demonstrate any membrane damage and alteration in the functional protein components, Figures 5 and 6, unlike our previous observations [28].

Multiphoton imaging in the transmission mode did not demonstrate any endothelial disruption in the VsEVH samples, Figure 1. Even though this observation may contradict our functional (calcium/nitric oxide) assays, it is not imperative to observe disrupted endothelium to observe attenuated function. Perhaps some stretching and/or manipulation of the vessel inherent in VsEVH technique were sufficient to temporarily impair endothelium function. There is also the possibility that cautery thermal effects and CO_2 _insufflation used in VsEVH techniques, though minimal with VirtuoSaph, could potentially be harmful to the vein and may be responsible for our observations. We are currently in the process of evaluating the effects of CO_2 _on vein structure and function to eliminate one possibility.

## Conclusion

The principal findings of this investigation is that unlike in our previous endoscopic harvesting study [28], extraction of the SV using the VirtuoSaph lends to preservation of morphological structure and biochemical function in the conduit. These results imply that it is not the EVH technique per se that causes conduit damage and eventual graft failures [26,27] but other pertinent factors may contribute to this problem. However, comparative studies are required to examine not only graft patency rates at one year postoperatively, but also patient outcomes with respect to the technique used to harvest the SV conduits. Irrespective of method of endoscopic harvest used, proper instrumentation, procedural training and technical expertise of the personnel involved in the process is of crucial importance to preserve the saphenous vein as a truly viable bypass conduit.

## Competing interests

The authors declare that they have no competing interests between the conclusions and authors.

## Authors' contributions

BEH: Study design, execute the experiment, data analyses; and help write the manuscript. XGL: Executing the experiments. JAW: Experimental design and collection of surgical samples and help editing the manuscript. HST: Designing and executing the experiments; writing and editing of the manuscript. The authors read and approved the manuscript
